# Analytical Study on Multi-Tier 5G Heterogeneous Small Cell Networks: Coverage Performance and Energy Efficiency

**DOI:** 10.3390/s16111854

**Published:** 2016-11-04

**Authors:** Zhu Xiao, Hongjing Liu, Vincent Havyarimana, Tong Li, Dong Wang

**Affiliations:** 1 College of Computer Science and Electronic Engineering, Hunan University, Changsha 410082, China; zhxiao@hnu.edu.cn (Z.X.); liuhognjing@hnu.edu.cn (H.L.); havincent14@hnu.edu.cn (V.H.); litong@hnu.edu.cn (T.L.); 2 State Key Laboratory of Integrated Service Networks, Xidian University, Xi’an 710071, China

**Keywords:** heterogeneous networks, small cells, energy efficiency, coverage performance

## Abstract

In this paper, we investigate the coverage performance and energy efficiency of multi-tier heterogeneous cellular networks (HetNets) which are composed of macrocells and different types of small cells, i.e., picocells and femtocells. By virtue of stochastic geometry tools, we model the multi-tier HetNets based on a Poisson point process (PPP) and analyze the Signal to Interference Ratio (SIR) via studying the cumulative interference from pico-tier and femto-tier. We then derive the analytical expressions of coverage probabilities in order to evaluate coverage performance in different tiers and investigate how it varies with the small cells’ deployment density. By taking the fairness and user experience into consideration, we propose a disjoint channel allocation scheme and derive the system channel throughput for various tiers. Further, we formulate the energy efficiency optimization problem for multi-tier HetNets in terms of throughput performance and resource allocation fairness. To solve this problem, we devise a linear programming based approach to obtain the available area of the feasible solutions. System-level simulations demonstrate that the small cells’ deployment density has a significant effect on the coverage performance and energy efficiency. Simulation results also reveal that there exits an optimal small cell base station (SBS) density ratio between pico-tier and femto-tier which can be applied to maximize the energy efficiency and at the same time enhance the system performance. Our findings provide guidance for the design of multi-tier HetNets for improving the coverage performance as well as the energy efficiency.

## 1. Introduction

Driven by the proliferation of fast developing wireless devices and the emergence of new services, the wireless and mobile data traffic has been approximately doubling every year and this growth is continuing unabatedly [1]. According to the prediction and statistical analysis from International Telecommunication Union (ITU) [2], a 1000-fold increase in wireless and mobile traffic is expected between 2010 and 2025, with a further 10–100 times growth in the period from 2020 to 2030. To address this exponential growth of mobile data traffic, various solutions are needed to meet the continuously increasing demand and offload traffic for the current cellular networks [3]. With the evolution to LTE and 5G, the cellular network has developed as a multi-tier network that comprises a conventional cellular network (i.e., macrocell network) with multiple low-power base stations (i.e., small cells) [4]. Massive use of small cells in such heterogeneous networks (HetNets), including picocells and femtocells overlaid on a macrocell network, is one of the promising techniques to cater for the ever increasing huge demand for future wireless data [5,6,7].

Nevertheless, the random deployment of a diverse set of small cells in HetNets makes resource management become increasingly complicated, because each tier of small cell base stations, e.g., the pico-tier and femto-tier, consisting of picocells and femtocells, respectively, is likely to have distinctly different characteristics [8,9]. The existing macrocell base stations (MBSs) are regularly deployed and with high transmission power such as to provide wide-range coverage and wireless access for the mobile users [9]. With a shorter communication range, picocells operate in the licensed spectrum with medium transmission power and offload traffic from the macrocell in hotspot areas [5,8]. Femtocells, which have very small coverage areas and much lower transmission power, are able to provide better quality of service (QoS) and higher data rates for their associated users [10]. In addition, the enormous number of running small cell base stations (SBSs) makes the energy consumption in the cellular network raise to a whopping value [11]. This in turn causes considerable increase in the cellular network operational burden and restricts the green development of heterogeneous small cell networks [12,13]. Therefore, the study of the performance enhancement and energy efficiency in HetNets is one of the key concerns, especially for the dense small cell scenarios.

Lots of research has focused on investigating the relationship between coverage performance and base stations’ density [14,15,16,17,18,19,20]. In [14], the system throughput for two-tier HetNets is verified by evaluating the areal capacity, and this is provided for co-channel scenario (CCS) as well as separate channel scenario (SCS) regarding the BS density. In [15], the authors investigate the aggregate interference statistics under several assumptions, such as always-on-BSs and uncorrelated interferers. The closed-form solutions of the average rate and optimal deployment density are derived for homogeneous interference-limited picocell networks. In [16], the authors study the relation between network capacity and the density of micro base stations in heterogeneous networks that consist of a macro base station and micro base stations. In [17], the authors investigate the capacity problem of heterogeneous two-tier wireless networks in four types of scenarios under a given delay constraint. The authors in [18] analyze the optimal BS density for both homogeneous and heterogeneous cellular networks with a service outage probability constraint. A small cell deployment scheme (SDS) is proposed in [19] to optimize the network throughput over time-varying distributions of users’ locations, in which the network throughput is improved by integrating the cooperation and resource allocation across small cells. One interesting finding in [14,15,16,17,18,19] is that the network capacity does not increase monotonously with the BSs’ density. According to the existing works [14,15,16,17,18,19], the interesting result is twofold: (i) the network capacity can be improved by deploying a large number of BSs in the traditional macrocell network; (ii) the network capacity would not keep increasing monotonously with the BSs’ density. Therefore, in our study, we investigate the internal links between coverage performance and small cell density and expand this problem to the scenario of multi-tier heterogeneous networks.

On the other hand, much research pays attention to energy efficiency in such dense small cell networks. A novel approach for joint power control and user scheduling is proposed in [20] for optimizing energy efficiency in terms of bits per unit energy consumption in the ultra-dense small cell networks (UDNs). In [21], the authors assume that all BSs in a small cell network form a homogeneous Poisson point process and propose all-on and on-off power control schemes (PCS) to obtain the average achievable cell rates for a given small cell density. The authors in [22] determined the associated tradeoffs of energy efficient cellular networks through the deployment of small cells. The success probability and energy efficiency in homogeneous macrocell (single-tier) and heterogeneous networks (two-tier) are both derived in [22], which confirms that the deployment of small cells leads to high energy efficiency, but this gain saturates as the density of small cells increases [23].

All those previous works on the system performance in a heterogeneous small cell network, in other words, how to characterize the coverage performance and energy efficiency and investigate the inherent relationship between them and the small cells deployment density, are still fairly minimal. In particular, all those previous works focus on addressing the small cell deployment problem for the two-tier HetNets which are formed of traditional macrocells overlaid by a single small cell tier such as picocells or femtocells. Moreover, there is a lack of consideration for the users’ load-heterogeneity that comes out of different small cell tiers.

In this paper, we lay emphasis on exploring the intrinsic links among coverage performance, energy efficiency and small cell density in the multi-tier heterogeneous networks. In this context, we propose an analytical framework for a multi-tier heterogeneous network with the purpose of obtaining the maximum energy efficiency and achievable throughput without substantially degrading the coverage performance in the meantime. Firstly, we use the baseline Poisson point process (PPP) for tractably modeling the multi-tier HetNets system [24] and we analyze the Signal to Interference Ratio (SIR) via studying the cumulative interference from each tier. By utilizing stochastic geometry tools, we then derive the analytical expressions of coverage probability and throughput for pico-tier as well as femto-tier. In order to evaluate the system performance, we devise the disjoint channel allocation scheme and study the system channel throughput for small cell tiers by taking user experience into consideration. Different from the existing works, we formulate the energy efficiency optimization problem under constraints of throughput and resource allocation fairness. To solve this problem, we devise a linear programming based method to generate the available area to obtain the feasible solutions. Through the system-level simulations, we present how the varying of system parameters affects coverage performance and energy efficiency. The comparative studies demonstrate that our proposed method is able to achieve better system performance when compared with existing methods. The optimal pico-femto density ratio for the dense small cells is verified, which can be used to guide the topological design for the multi-tier HetNets.

The remainder of the paper is organized as follows: Section 2 presents the modeling of the multi-tier HetNets and the SIR analysis. Section 3 derives the coverage probability to study the coverage performances for pico-tier and femto-tier respectively. Section 4 devises the channel allocation scheme and defines the system throughput for different tier. Section 5 formulates the energy minimization problem and proposes its solution based on linear programming. Section 6 provides numerical results based on the system-level simulations. Finally, Section 7 concludes the paper.

## 2. Modeling of Multi-Tier Heterogeneous Networks

**Figure 1:**
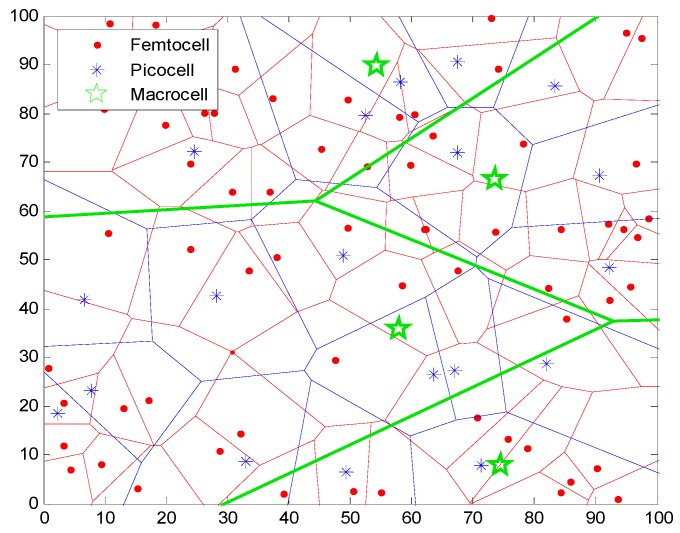
Base stations in multi-tier HetNets drawn from a PPP distribution include macrocells and different types of small cells, namely in this study, picocells and femtocells.

According to [25], a concise and tractable model begins with a spatial point process to statistically model the base station locations in HetNets. The simplest and best known such point process, namely the Poisson point process (PPP) [26], assumes that base stations in each small cell tier are independently distributed with density *λs*. In this study, we consider a multi-tier heterogeneous network consisting of macrocells and two different types of small cells, picocell and femtocell, which constitutes pico-tier and femto-tier, respectively. As shown in Figure 1, the PPP distribution of macrocells and small cells are illustrated. Picocell base stations (PBSs) are deployed to enhance the coverage of a macrocell for a wide range whose coverage radius is *R_p_*. Femtocell base stations (FBSs) are used for homes, offices and other personal area that have high speed traffic demands. Let *R_f_* denote the coverage radius of FBS and we have *R_p_* > *R_f_*. Hence, in the pico-tier, we can assume the PBS deployment distribution follows PPP of density *λ_p_*. The picocell users’ distribution follows PPP of density *μ_p_*. In order to obtain the best link quality, each picocell user accesses the nearest PBS as the serving base station. The distance between them is expressed as *r_p_*. Thus, according to the properties of PPP, the probability density function (PDF) of *r_p_* can be given by:

(1) $$f_{pico}\left(r_{p}\right)=2\pi \lambda_{p}r_{p}\;\text{exp}(-\lambda_{p}\pi {r_{p}}^{2})$$

where $r_{p}\in \left(0,R_{p}\right)$.

In the femto-tier, the FBS deployment distribution follows PPP of density *λ_f_*. It is noteworthy that femtocells have very small coverage areas and much lower transmission power while providing better quality of service (QoS) and higher data rates for their associated users. Moreover, picocell users and femtocell users have different wireless traffic load demands. Therefore, we use different users’ distributions in order to describe load-heterogeneity of multi-tier heterogeneous networks. To this end, we assume that the femtocell user distribution follows a uniform distribution. Each femtocell user accesses the nearest FBS as the serving base station to ensure the best service experience. The distance between them is represented as *r_f_*. Therefore, based on probability theory, the PDF of *r_f_* can be expressed as [27]:

(2) $$f_{femto}\left(r_{f}\right)=2r_{f}/{R_{f}}^{2}$$

where $r_{f}\in \left(0,R_{f}\right)$.

The standard power loss propagation model is applied in our system model and the path loss exponent is set to α > 2 [28]. We assume that Rayleigh fading channel [29,30] exists between the user and the service base station, i.e., *h*~exp(1). Therefore, the signal power that the user receives from its serving base station can be calculated as *Phr^−α^*, where *r* represents the distance from the serving base station to the user, *P* represents the transmit power of the serving base station. Consider that the user is served by the small cell base station (SBS), i.e., femtocell base station or picocell base station in this study, the Signal to Interference plus Noise Ratio (SINR) of the user can be given as:

(3) $$SINR=\frac{P_{S}hr^{-\alpha }}{I_{r}+\delta^{2}}$$

where *P_S_* is the transmit power of the small cell base station and *δ* denotes the noise power. In Equation (3), *I_r_* is used to represent the cumulative interference power, which denotes the sum of the power that the user receives from all interfering base stations [31]. *I_r_* can be given by:

(4) $$I_{r}=\sum_{i\in \Phi }P_{S}g_{i}^{S}R_{i}^{-\alpha }+\sum_{j\in \Theta }P_{M}g_{j}^{M}R_{j}^{-\alpha }$$

where, Φ represents the set of all interfering SBSs located in the same tier and Θ stands for the set of the macrocells; *P_M_* is the transmit power of the macrocell base station. *R_i_* and *R_j_* are the distance from the user to the *i*-th SBS and *j*-th MBS respectively and *R_i_* > *r* is assumed. $g_{i}^{S}$ and $g_{i}^{M}$ are the channel gain of the *i*-th SBS and *j*-th MBS, respectively.

In [21] the authors proved that inter-cell interference is the main factor affecting the network performance. The thermal noise does not have a significant impact on the network performance. In addition, compared to the signal power and the interference power separately, the thermal noise power is smaller enough to be neglected. Thus, the thermal noise is not considered in our system model. Hence, we use Signal to Interference Ratio (SIR) instead of SINR and Equation (3) can be rewritten as:

(5) $$SIR=\frac{P_{S}hr^{-\alpha }}{I_{r}}$$

## 3. Coverage Performance

Without loss of generality, the SIR threshold *T* is set for each tier in order to guarantee different tiers’ service quality [32,33]. The user is associated with the service base station if SIR is higher than *T*, which means that the user can access the tier successfully; on the contrary, the user is not in the coverage of the service base station once his current SIR is lower than *T*. This is the fundamental concept with which one can conduct statistical analysis on the coverage performance for the cellular networks. On the basis of this principle, which is adopted in [16,26,32,33], we obtain the definition of coverage probability in this section. Besides, we derive Lemmas 1 and 2 such as to give analytical expressions of coverage performance for multi-tier HetNets, namely pico tier and femto tier in our study.

**Definition 1** (Coverage probability)**.** *The average coverage probability of the user is considered as the conditional probability that the user can access to the SBS, which is defined as:*

(6) q=E[p(SIR>T|r)]

### 3.1. Coverage Performance of Pico-Tier

**Lemma** **1.** *The average coverage probability of the picocell user, which is denoted by q_p_, can be expressed as:*

(7) $$q_{p}=\int_{0}^{R_{p}}L_{I_{r}}\left(T_{P}r^{\alpha }/P_{P}\right)2\pi \lambda_{P}r\;\text{exp}\left(-\lambda_{P}\pi r^{2}\right)dr$$

*where, T_p_ is the SIR threshold of the pico-tier, P_p_ is the transmission power of the picocell base station (PBS),* $L_{I_{r}}\left(\cdot \right)$ *denotes the Laplace transform.*

**Proof.**  According to Equation (6), in the pico-tier, the average coverage probability of a picocell user, which is denoted by *q_p_*, can be given by:

(8) $$q_{p}=E\left[p\left(SIR>T_{p}|r_{p}\right)\right]$$

Based on Equation (5), *q_p_* in Equation (8) can be rewritten as:

(9) $$q_{p}=E\left(p\left[\left(hP_{p}r_{p}^{-\alpha }/I_{r}\right)>T_{p}|r_{p}\right]\right)$$

By making use of the statistical distribution of *r_p_* and the definition of mathematical expectation, Equation (7) can be further transformed into the following expression:

(10) $$\begin{array}{ll} q_{P} & =\int_{0}^{R_{p}}p\left(\frac{P_{P}hr_{p}^{-\alpha }}{I_{r}+\delta^{2}}>T_{P}|r_{p}\right)\cdot 2\pi \lambda_{P}r_{p}\;\text{exp}\left(-\lambda_{P}\pi r_{p}^{2}\right)dr\\ & =\int_{0}^{R_{p}}p\left(h>T_{P}r_{p}^{\alpha }I_{r}/P_{P}|r_{p}\right)\cdot 2\pi \lambda_{P}r_{p}\;\text{exp}\left(-\lambda_{P}\pi r_{p}^{2}\right)dr \end{array}$$

Due to *h*~exp(1) [30], $p(h)>\left(T_{P}r_{p}^{\alpha }I_{r}\right)/\left(P_{P}|r_{p}\right)$ in Equation (10) can be further deduced as:

(11) $$\begin{array}{cc} p\left(h>T_{P}r_{p}^{\alpha }I_{r}/P_{P}|r_{p}\right) & =E_{r_{p}}\left(\text{exp}\left[-T_{p}r_{p}^{\alpha }I_{r}/P_{P}|r_{p}\right]\right)\\ & =L_{I_{r}}\left(S\right){|}_{S=T_{P}r_{p}^{\alpha }/P_{P}} \end{array}$$

This completes the proof. ☐

Here, ${ℒ}_{I_{r}}{\left(S\right)}_{S=T_{P}r^{\alpha }/P_{P}}$ represents the Laplace transform of the cumulative interference power *I_r_* that the picocell user receives. The deduction for ${ℒ}_{I_{r}}{\left(S\right)}_{S=T_{P}r^{\alpha }/P_{P}}$ can be found in Appendix A.

### 3.2. Coverage Performance of Femto-Tier

**Lemma** **2.** *The average coverage probability of the femtocell user, which is denoted by q_f_ and can be expressed by:*

(12) $$q_{f}=\int_{0}^{R_{f}}\text{exp}\left[\frac{-2\pi^{2}\lambda_{f}T_{f}^{2/\alpha }r_{f}^{2}}{\alpha \text{sin}\left(2\pi /\alpha \right)}\right]2r_{f}/R_{f}^{2}dr_{f}$$

**Proof.**  According to Equation (6), in the femto-tier, the average coverage probability of a femtocell user can be defined as:

(13) $$q_{f}=E\left[p\left(SIR>T_{f}|r_{f}\right)\right]$$

Based on Equation (2), taking advantage of the statistical distribution of *r_f_* and the definition of mathematical expectation, Equation (13) can be further expressed as the following expression:

(14) $$\int_{0}^{R_{f}}p\left(SIR>T_{f}|r_{f}\right)f_{r_{f}}\left(r_{f}\right)dr_{f}=\int_{0}^{R_{f}}p\left(SIR>T_{f}|r_{f}\right)2r_{f}/R_{f}^{2}dr_{f}$$

Recall the femto-tier follows a uniform distribution, we utilize the derived result for P(*SIR*>*T_f_*|*r_f_*) from [18], which can be expressed by:

(15) $$p\left(SIR>T_{f}|r_{f}\right)=\text{exp}\left[\frac{-2\pi^{2}\lambda_{f}T_{f}^{2/\alpha }r_{f}^{2}}{\alpha \text{sin}\left(2\pi /\alpha \right)}\right]$$

Taking Equation (15) into Equation (14) gives the result Equation (12). This completes the proof. ☐

## 4. Channel Allocation and Throughput

This section defines the throughput for the pico-tier and the femto-tier, respectively, in order to facilitate the subsequent establishment of system energy efficiency optimization.

### 4.1. Channel Allocation

Without loss of generality, the total system bandwidth is set to be *W*. A fixed number of consecutive subcarriers compose a system channel whose bandwidth is *w*. Therefore, the total number of channels used in the multi-tier HetNets can be expressed as *W*/*w*. **B** = {*w*_1_, *w*_2_, *w*_3_,…, *w_n_*,…, *w_W/w_*} is a set which is composed of the total system channels. Let **B**_*p*_ and **B**_*f*_ denote the channels used by the pico-tier and femto-tier, respectively. We have **B**_*p*_,**B**_*f*_ ⊆ **B** and **B**_*p*_ ∪ **B**_*f*_ = **B**. In order to eliminate the cross-tier interference between the pico-tier and the femto-tier, we assume **B**_*p*_ ∩ **B**_*f*_ = *φ* and **B**_*p*_,**B**_*f*_ ≠ *φ*. Therefore, the coverage performance and the throughput performance of each tier rely only on the channels used by the corresponding tier, which is no longer affected by the channel used by other tiers in the multi-tier HetNets.

### 4.2. System Throughput

**Definition 2** (System channel throughput)**.** *The system channel throughput is defined as the throughput on any channel w_n_*, *n* ∈ {1,2,3,…, *W*/*w*}*, which can be given by:*

(16) $$\Delta \left(w_{n}\right)=q\;\text{log}(1+T)$$

*The throughput of the pico-tier is defined as:*

(17) $$\begin{array}{ll} \Delta_{p} & =\lambda_{p}\sum_{w_{p}\in {\text{Β}}_{p}}\Delta \left(w_{p}\right)\\ & =\lambda_{p}\left(\left|{Β}_{p}\right|q_{p}\;\text{log}(1+T_{p})\right) \end{array}$$

*where w_p_ represents the channel used by the pico-tier.*

*Based on Definition 2, the throughput of the femto-tier is defined as:*

(18) $$\begin{array}{ll} \Delta_{f} & =\lambda_{f}\sum_{w_{f}\in {\text{Β}}_{f}}\Delta \left(w_{f}\right)\\ & =\lambda_{f}\left(\left|{Β}_{f}\right|q_{f}\;\text{log}(1+T_{f})\right) \end{array}$$

*where w_f_ represents the channel used by the femto-tier.*

*In conclusion, the throughput of the system model can be expressed as:*

(19) $$\Delta_{s}=\Delta_{p}+\Delta_{f}$$

## 5. Energy Efficiency Optimization

### 5.1. Fairness of Channel Allocation

We can see that according to Equations (17) and (18), no matter whether a pico-tier or femto-tier, the throughput of each tier increases linearly with the number of channels used by the tier. This shows that if the fairness of channel allocation is not taken into account when allocating channel resources for each tier, there could be large differences in the number of channels assigned to different tiers [34,35]. In addition, this will lead to situations where a certain tier has far more channels than other tiers. As a result, the user experience of this tier is much better than in other tiers, while the user experience of other tiers is relatively low. Therefore, in order to avoid the unfairness of channel allocation, we establish the following fairness of channel allocation from the perspective of the throughput performance of each tier:

(20) $$\varepsilon \leq \frac{\left|{Β}_{p}\right|q_{p}\;\text{log}(1+T_{p})}{\left|{Β}_{f}\right|q_{f}\;\text{log}(1+T_{f})}\leq \ell$$

where the upper ℓ and lower bounds *ε* are the pre-set variables.

### 5.2. Optimization of Energy Efficiency

Based on the channel allocation fairness, the optimization problem of system energy efficiency maximization is formulated to explore how the SBS deployment density affects the system energy efficiency.

**Definition 3** (Energy efficiency)**.** *The system energy efficiency is defined as the ratio of the system throughput to the power consumption per unit area within the system model, which can be given by:*

(21) $$\eta_{EE}=\frac{\Delta_{s}}{\lambda_{p}P_{p}+\lambda_{f}P_{f}}$$

*Then, the energy efficiency maximization problem of the system model can be induced to the following optimization problem:*

(22) $$\underset{\left|{Β}_{p}\right|,\left|{Β}_{f}\right|}{\text{max}}\eta_{EE}$$

(23) $$s.t.\;\frac{\left|{Β}_{p}\right|q_{p}\;\text{log}(1+T_{p})}{\left|{Β}_{f}\right|q_{f}\;\text{log}(1+T_{f})}\leq \ell$$

(24) $$\frac{\left|{Β}_{p}\right|q_{p}\;\text{log}(1+T_{p})}{\left|{Β}_{f}\right|q_{f}\;\text{log}(1+T_{f})}\geq \varepsilon$$

(25) $$\left|{Β}_{p}\right|+\left|{Β}_{f}\right|\leq \left|Β\right|$$

**Figure 2:**
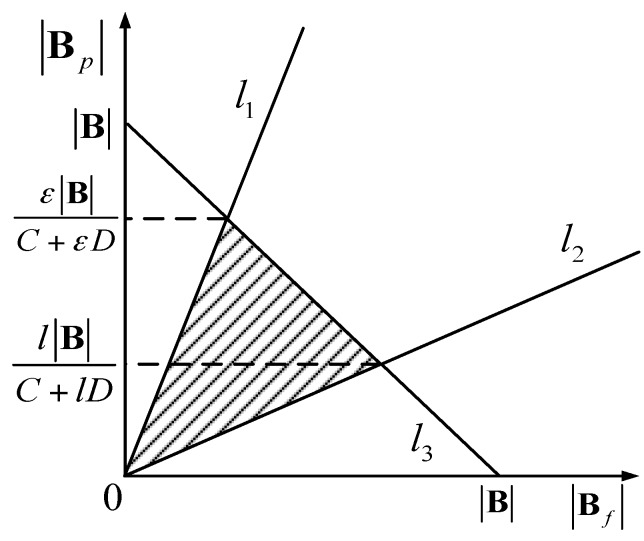
Available area for Equations (22)–(25).

We propose a linear programming-based approach to derive an available area in order to solve the problem in Equation (22) with low computional complexity. The constraints of throughput performance and resource allocation fairness are shown in Figure 2, in which lines *l*_1_, *l*_2_ and *l*_3_ represent Equations (23)–(25), respectively. In Figure 2, for the sake of simplicity, we let C and D stand for *q_p_*log(1 + *T_p_*) and *q_f_*log(1 + *T_f_*), respectively. The area on the right of *l*_1_ and above *l*_2_ is where the minimum figure is satisfied.

Although we design a linear programming-based approach for the proposed optimization problem, channel allocation and throughput leading to solve the optimization problem in closed form is quite difficult due to the non-trivial relations between the small cells’ deployment. It is noteworthy that the macrocells and small cells are randomly distributed in the multi-tier HetNets. In addition, the small cells’ users are located within their serving SBS in a random manner. For instance, the location of femtocell users is random and follows a uniform distribution. Based on Figure 2, we mainly focus on the avalaible area where the optimal solution is more likely to appear. It implies we can find the optimal solution with numerical calculations. Therefore, in the next section, we implement numerical simulations to explore how the small cells coverage performance and system energy efficiency vary with the SBS deployment density ratio and other parameters.

## 6. Simulation Results and Analysis

### 6.1. Simulation Setup

In this section, we evaluate the performance of multi-tier heterogeneous networks and assess how the coverage performance and the energy efficiency change with the small cells’ deployment density. The distributions of macrocells, picocells and femtocells are independently distributed and follow the Poisson point process (see Figure 1). We do the simulations by Matlab2013a and numerical simulation results are computed after averaging 1000 independent runs. We use the generic WINNER II channel model [36] in the simulation to obtain reliable results. The key simulation parameters are set up according to [37] and are given in Table 1. The path loss models are based on [38]. The pre-set variables ℓ and lower bounds *ε* are set to 0.2 and 0.8, respectively.

### 6.2. Discussion and Analysis Results

**Figure 3:**
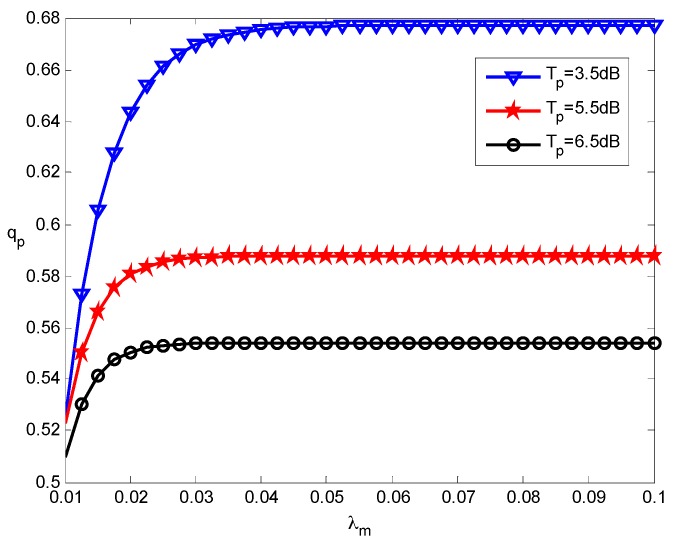
Effect of picocell density on the pico-tier coverage performance.

We first look into the coverage performance of the pico-tier and femto-tier with various deployment densities. In the simulation, we obtain the numerical results of coverage probabilities by using Lemmas 1 and 2. Figure 3 shows the coverage probability of pico-tier varies with different picocell densities and various SIR threshold values. With the growth of the picocell density *λ_p_*, the picocell users’ average coverage probability *q_p_* increases under different SIR thresholds *T_p_*. The reason behind this is that the picocell users are closer to their associated picocells when there are more picocells deployed. Hence, it is more likely that the received SIR at the picocell users can exceed the pico-tier SIR threshold (namely *T_p_*). As a result, the coverage performance is improved. We can also see that for each choice of *T_p_*, *q_p_* converges to a stable value when *λ_p_* keeps increasing. This is because the inter-picocell interference will be more severe as*λ_p_* increases. In addition, it can be seen that *q_p_* drops when enlarging *T_p_* under the same *λ_p_*, in other words, the coverage performance of the pico-tier declines. This is because it is more difficult for users to access pico-tiers when the pico-tier SIR threshold becomes larger.

**Figure 4:**
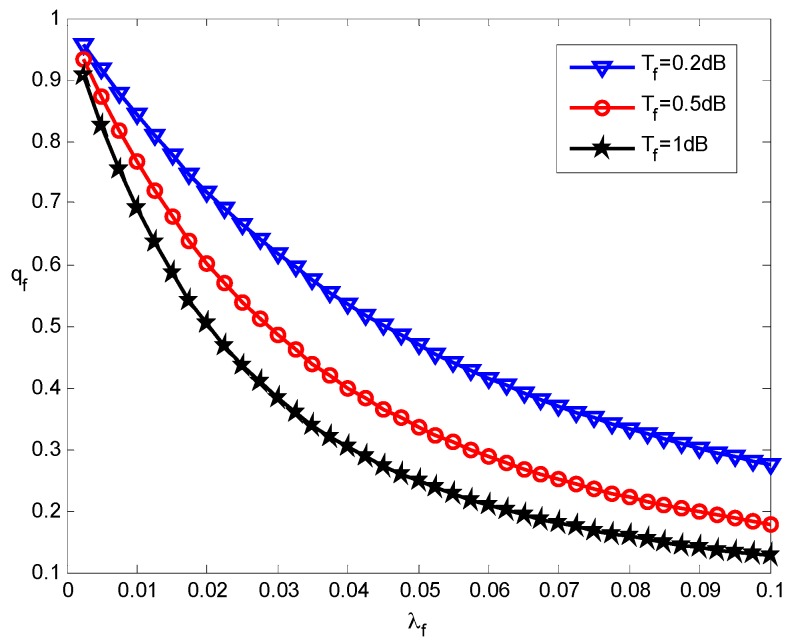
Effect of femtocell density on the femto-tier coverage performance.

Figure 4 presents the relationship between the coverage performance of femto-tier and the femtocell deployment density under the various SIR thresholds. We see that the femtocell users’ average coverage probabilities *q_f_* decrease as the femtocell deployment density *λ_f_* increases, which is contrary to the case of pico-tiers. That is because that increasing *λ_f_* will cause more severe intra-femto-tier interference and hence this decreases coverage performance in the femto-tier. Moreover, for a given *λ_f_*, *q_f_* decreases when the femto-tier SIR threshold *T_f_* grows. These results from the fact that using a larger *T_f_* would degrade the coverage performance of the femto-tier, which is consistent with the pico-tier results (see Figure 3). According to the results from Figure 3 and Figure 4, the interference-limited effect appears when the density of small cells increases. Therefore, by increasing *λ_p_* (or *λ_f_*) will cause more considerable inter-small cells interference and this in turns results in an overall decrease of the coverage performance.

**Figure 5:**
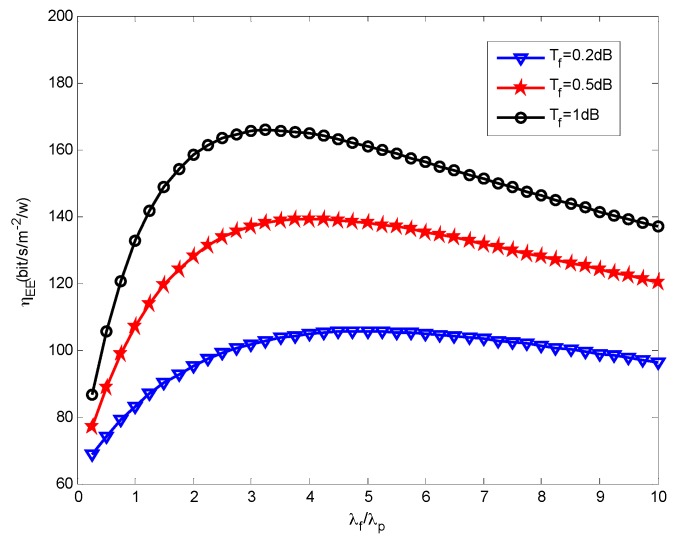
Energy efficiency vs. SBS density ratio when *T_p_* = 3.5 dB.

**Figure 6:**
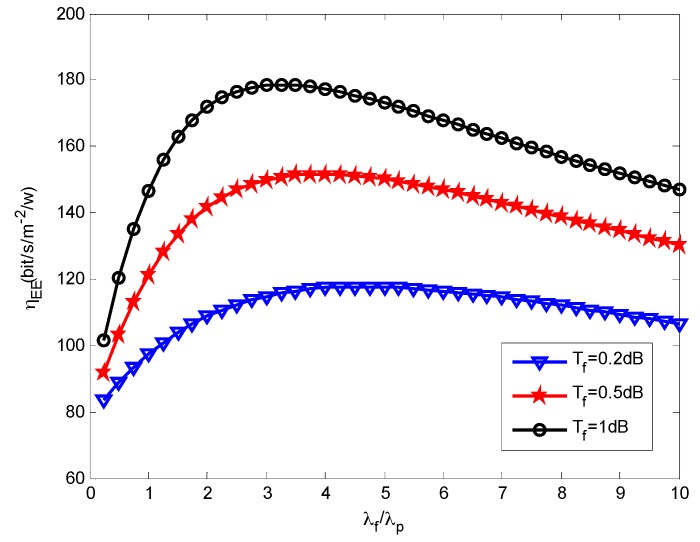
Energy efficiency vs. SBS density ratio when *T_p_* = 5.5 dB.

**Figure 7:**
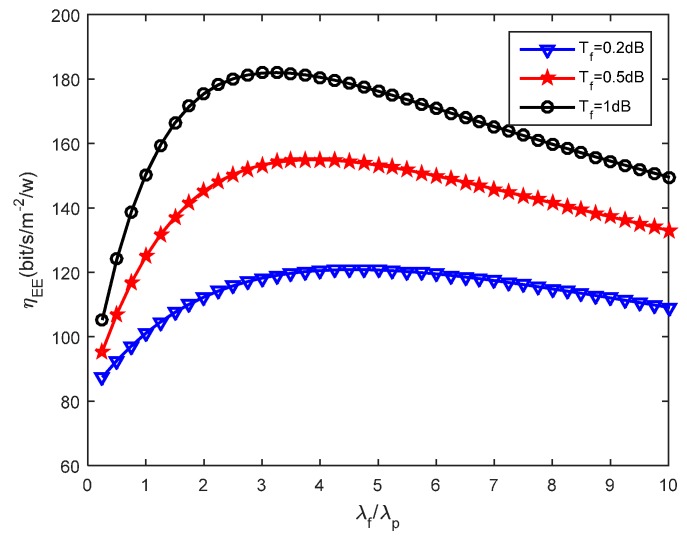
Energy efficiency vs. SBS density ratio when *T_p_* = 6.5 dB.

We then assess how the small cells density affects the energy efficiency. Figure 5, Figure 6 and Figure 7 present the effects that the picocell and femtocell deployment density ratio has on the system energy efficiency under the various SIR thresholds. In these simulations, we set the picocell deployment density *λ_p_* = 0.01/m^2^. It can be found out that the energy efficiency *η_EE_* rises rapidly to a peak value and then decreases with the increasing deployment density ratio *λ_f_*/*λ_p_*. This proves that deploying the femtocells can improve the energy efficiency to some extent under varying picocell deployment density conditions. Nevertheless, the energy efficiency can be reduced when the femtocell deployment density exceeds a certain value. According to Definition 3 (see Section 5.2), this is because excessive femtocell deployment will cause serious inter-cell interference and result in a gradual decrease of the coverage performance, which thus determinates the throughput of femto-tier.

Moreover, we can observe the growth of *η_EE_* with the increase of *T_f_* under a given *T_p_*. For example, we can look at the results from Figure 6 and Figure 7, in which *T_p_* is set to 5.5 dB and 6.5 dB, respectively. The larger *T_f_* will decrease the coverage performance because it will hinder more users from accessing femtocells, which is supported based on the results from Figure 4. However, it brings a benefit for the users that are able to access the femto-tier, in other words, these users, which can receive rather good SIR, will have more channel resources. Therefore, the throughput in the femto-tier is improved. In addition, by comparing the results in Figure 5, Figure 6 and Figure 7, we can see that *η_EE_* increases with the increase of *T_p_*. This is because with the increase of the pico-tier SIR threshold, the accessed picocell users can be assigned more channel resources. As a consequence, this improves the throughput of picocell users and enhances the energy efficiency.

**Figure 8:**
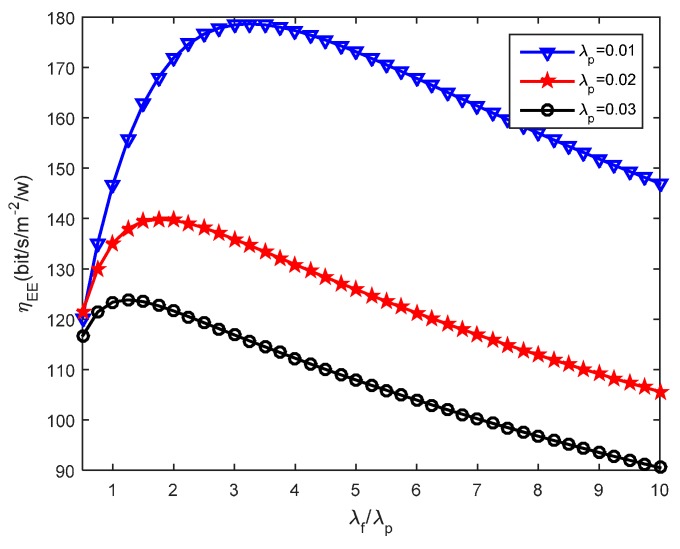
Energy efficiency under different picocell deployment density when *T_p_* = 5.5 dB and *T_f_* = 1.0 dB.

**Figure 9:**
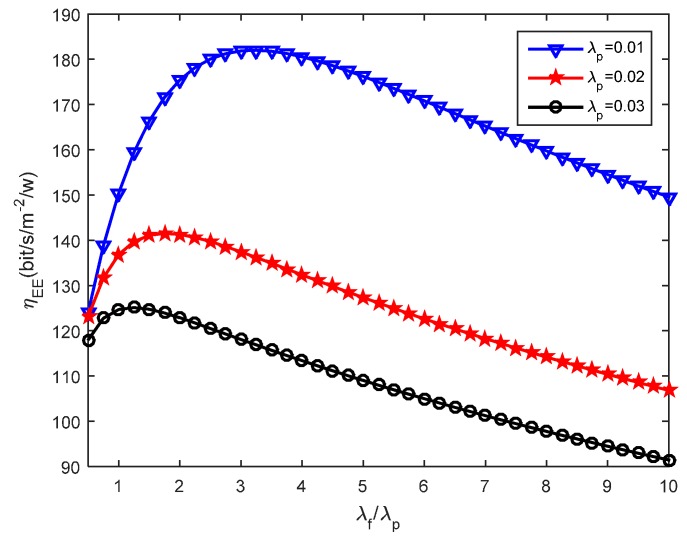
Energy efficiency under different picocell deployment density when *T_p_* = 6.5 dB, *T_f_* = 1.0 dB.

**Figure 10:**
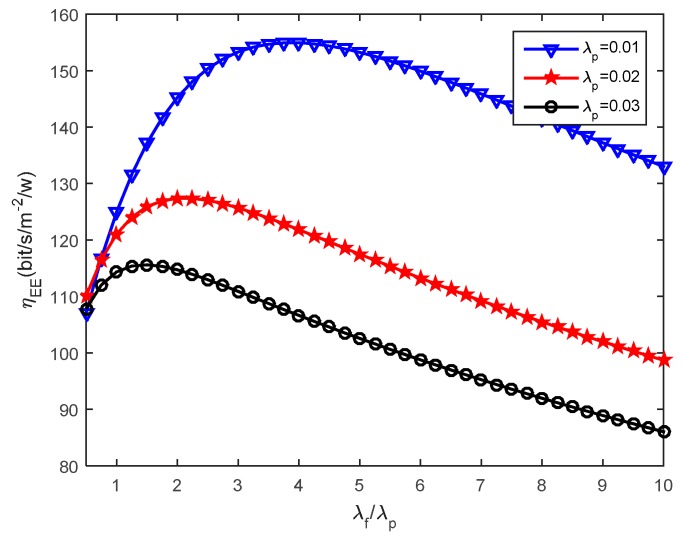
Energy efficiency under different picocell deployment density when *T_p_* = 6.5 dB and *T_f_* = 0.5 dB.

Furthermore, we study how the system energy efficiency varies with the femtocell and picocell deployment density ratio with different picocell deployment densities. Figure 8, Figure 9 and Figure 10 present the impact of small cells’ density ratio on the energy efficiency under various SIR requirements. In Figure 8, three curves can be seen which indicate that the system energy efficiency *η_EE_* increases first and then decreases with the increasing small cell density ratio *λ_f_*/*λ_p_* when the picocell deployment density *λ_p_* is set to 0.01, 0.02 and 0.03, respectively. Consistent results can be found in Figure 9 and Figure 10. Hence, the number of femtocells should not be much larger than the number of picocells, otherwise, this will cause severe intra femto-tier interference and result in significant energy consumption, which thus decreases the system energy efficiency. When studying the simulation results under different values of *T_p_* and *T_f_* , it can be found that better energy efficiency is obtained with larger *T_p_* and *T_f_*. For example, *η_EE_* in Figure 9 is better than that in Figure 10, since *T_f_* is set to 1.0 dB in Figure 9 while *T_f_* is 0.5 dB in Figure 10. This conclusion is also validated by the results in Figure 5, Figure 6 and Figure 7. In particular, we can see that there is always an optimal deployment density ratio *λ_f_*/*λ_p_* for each choice of *λ_p_* when various values of *T_p_* and *T_f_* are used, for example *T_p_* = 5.5 dB, *T_f_* = 1.0 dB and *T_p_* = 6.5 dB, *T_f_* = 0.5 dB in Figure 8 and Figure 10, respectively. The system energy efficiency is able to reach the maximum value when the optimal deployment density ratio is satisfied. We have tried different values of *λ_f_* and*λ_p_*, for example, when *λ_f_* and *λ* are multiplied by 2, as a reviewer suggested, we obtained similar curves. In these cases, we can also achieve the peak values of system energy efficiency while with different value of the maximum energy efficiency. Therefore, the existence of the optimal deployment density ratio implies that the system energy efficiency can be optimized by adjusting the small cells deployment density in different network tier.

Recall in Equation (22), we proposed the energy efficiency maximization problem, namely, to maximize *η_EE_* , which is highly related with the parameters *λ_p_* and *λ_f_*. The constraints, for instance, in Equations (23) and (24), rely on the parameters *λ_p_*, *λ_f_*, and the SIR thresholds *T_p_*, *T_f_*. In this study, we investigate the effects the picocell and femtocell deployment density ratio has on the energy efficiency under the various SIR thresholds. For example, from Figure 5, Figure 6 and Figure 7, we find out the growth of *η_EE_* will increase with the increasing of *T_f_* under a given *T_p_*. Moreover, by solving the optimization problem in Equation (22), we can obtain an optimal deployment density ratio *λ_f_*/*λ_p_* for each choice of *λ_p_* with respect to various values of *T_p_* and *T_f_*, these results are validated in Figure 8, Figure 9 and Figure 10. Moreover, we find out that system energy efficiency can be significantly improved and the optimal deployment density ratio increases as the picocell deployment density *λ_p_* decreases. For instance in Figure 9, the maximum *η_EE_* exceeds 180 bps/m^−2^/W when *λ_f_*/*λ_p_* is between 3 to 4 and *λ_p_* is set to 0.01, while in the case of *λ_p_* = 0.02, the maximum is 140 bps/m^−2^/W and *λ_f_*/*λ_p_* is less than 2. In a word, these simulations indicate that appropriately reducing the number of picocells to a low level e.g., *λ_p_* is less than 0.02, and in the meantime applying modest increase of femtocells is capable of offering an effective approach to improve the energy efficiency of the heterogeneous small cell networks.

**Figure 11:**
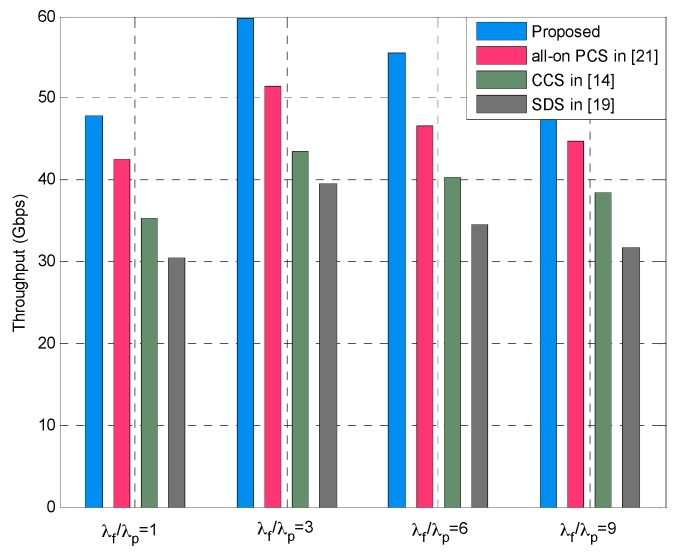
Overall throughput of small cells under different SBS density ratios when *T_p_* = 5.5 dB, *T_f_* = 1.0 dB.

**Figure 12:**
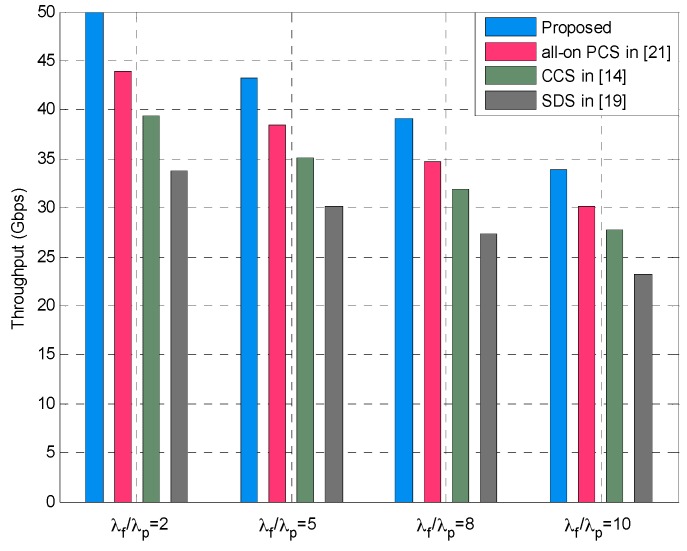
Overall throughput of small cells under different SBS density ratios when *T_p_* = 6.5 dB and *T_f_* = 0.5 dB.

In addition, to evaluate the system performance of our proposed method, we conduct simulations and comparative analysis with other methods. The SCS in [14], small cell deployment scheme (SDS) in [19] and all-on power control scheme (PCS) in [21] are applied to multi-tier HetNets and compared with the proposed approach in terms of the overall throughput in small cell tier. In Figure 11, the simulation results are obtained under various small cells density ratios, namely, *λ_f_*/*λ_p_* = 1, *λ_f_*/*λ_p_* = 3, *λ_f_*/*λ_p_* = 6, and *λ_f_*/*λ_p_* = 9, respectively. It can be seen that the proposed method outperforms the other methods and achieves best throughput of small cells under different values of *λ_f_*/*λ_p_*. When comparing the results in the cases *λ_f_*/*λ_p_* = 1 and *λ_f_*/*λ_p_* = 3, it should be noted that throughput increases because the number of femtocells increases as the SBS density ratio grows. We also observe that the overall throughput in the case of *λ_f_*/*λ_p_* = 3 outperforms the other three cases, which is due to the optimal SBS density ratio in such a case, which is also supported by the results in Figure 5 and Figure 6. In the cases of larger SBS density ratios, for instance, *λ_f_*/*λ_p_* = 6 and *λ_f_*/*λ_p_* = 9, we can observe the decrease of the overall throughput. The reason behind this is that increasing *λ_f_*/*λ_p_* means more femtocells are deployed in the multi-tier HetNets, and this consequently generates more severe intra-femto-tier interference. In other words, the interference-limited effect becomes significant, which degrades the coverage performance as well as the throughput (also see Figure 4). Similar results can be found from Figure 12 which depicts the throughput of small cells under four SBS density ratio cases, i.e., *λ_f_*/*λ_p_* = 2, *λ_f_*/*λ_p_* = 5, *λ_f_*/*λ_p_* = 8, and *λ_f_*/*λ_p_* = 10, respectively. It can be also seen that our proposed method is superior to the compared methods, and the throughput of small cells decreases gradually with the increasing *λ_f_*/*λ_p_*. The best throughput is obtained when *λ_f_*/*λ_p_* = 2, which is the optimal SBS density ratio in such a case and verified from the result in Figure 10.

## 7. Conclusions

In this paper, we propose a novel framework for multi-tier heterogeneous networks in order to characterize the coverage performance and energy efficiency and investigate the inherent relationship between them and the small cells deployment density. Based on PPP and statistical geometry theory, we derive the analytical expressions of the success probabilities for pico- and femto-tier after studying the cumulative interference from each tier. We design a disjoint sub-channel allocation scheme and investigate how to evaluate the throughput performance of various tiers. Then the energy efficiency optimization problem is formulated in terms of the throughput performance and the resource allocation fairness. To solve this optimization problem, we devise a linear programming based method to generate the available area for obtaining the feasible solutions. System-level simulation results show that SBS deployment density has a significant effect on the coverage performance and energy efficiency in different tiers. An optimal pico-femto density ratio is identified numerically that can be used to maximize the energy efficiency of the multi-tier HetNets and at the same time guarantee the coverage performance and system throughput.

We conclude that, to deliberately deploy the number of picocells i.e., *λ_p_* < 0.02, and in the meantime applying modest increase of femtocells, e.g., the density ratio *λ_f_*/*λ_p_* is 3 to 4, is proved to be an effective way to enhance the energy efficiency of the heterogeneous small cell networks. Therefore, this study should be quite helpful for designing the future dense small cells networks and provide guidance on improving the performance of HetNets by adjusting the small cells’ deployment density. Our future work involves the design of a multi-tier HetNets suitable for mobile scenarios, such as cross-tier handover, access admission, as well as mobility management.

## Figures and Tables

**Table 1:** Simulation parameters.

Parameter	Value
BSs distribution	PPP [24,26]
*P_M_*	46 dBm
*P_p_*	38 dBm
*P_f_*	24.8 dBm
*α*	4
System carrier	2 GHz
System bandwidth	40 MHz
Path loss for macrocell cellular link	26 log_10_(d[m]) + 39 + 20 log_10_(*f*_c_[GHz]/5.0)
Path loss for Picocell cellular link	22.7 log_10_(d[m]) + 41 + 20 log_10_(*f*_c_[GHz]/5.0)
Path loss for Femtocell cellular link	18.7 log_10_(d[m]) + 46.8 + 20 log_10_(*f*_c_[GHz]/5.0)

## Appendix A

We present the deduction for ${ℒ}_{I_{r}}{\left(S\right)}_{S=T_{P}r^{\alpha }/P_{P}}$. In Equation (11), ${ℒ}_{I_{r}}{\left(S\right)}_{S=T_{P}r^{\alpha }/P_{P}}$ is written as:

(A1) $$L_{I_{r}}\left(S\right)=E_{I_{r}}\left(e^{-SI_{r}}\right)$$

According to Equation (4), ${ℒ}_{I_{r}}{\left(S\right)}_{S=T_{P}r^{\alpha }/P_{P}}$ can be expressed as:

(A2) $$L_{I_{r}}\left(S\right)=E_{I_{r}}\left\{\text{exp}\left[-S\left(\sum_{i\in \Phi }P_{S}g_{i}^{S}R_{i}^{-\alpha }+\sum_{j\in \Theta }P_{M}g_{j}^{M}R_{j}^{-\alpha }\right)\right]\right\}$$

Then we have:

(A3) $$L_{I_{r}}\left(S\right)=E_{\Phi ,g_{i}}\left[\text{exp}\left(-S\sum_{i\in \Phi }P_{S}g_{i}^{S}R_{i}^{-\alpha }\right)\right]E_{\Theta ,p_{j}}\left[\text{exp}\left(-S\sum_{j\in \Theta }P_{M}g_{j}^{M}R_{j}^{-\alpha }\right)\right]$$

Based on the probability generating functional (PGFL) [39] of the PPP, we can obtain:

(A4) $$L_{I_{r}}\left(S\right)=\text{exp}\left[-2\pi \lambda_{P}\int_{r}^{\infty }\left(1-E_{g}\left(e^{-Sg^{S}v^{-\alpha }}\right)vdv\right)\right]\cdot \text{exp}\left[-2\pi \lambda_{M}\int_{r}^{\infty }\left(1-E_{p}\left(e^{-Sg^{M}v^{-\alpha }}\right)vdv\right)\right]$$

Since *g^S^*~exp(1) and *g^M^*~exp(1), then

(A5) $$L_{I_{r}}\left(S\right)=\text{exp}\left\{\begin{array}{l} \left[-2\pi \lambda_{M}\int_{0}^{\infty }\left(\int_{r}^{\infty }\left(1-e^{-Sgv^{-\alpha }}\right)vdv\right)f\left(g\right)dg\right]\\ -\left[2\pi \lambda_{S}\int_{0}^{\infty }\left(\int_{r}^{\infty }\left(1-e^{-Sqv^{-\alpha }}\right)vdv\right)f\left(q\right)dq\right] \end{array}\right\}$$

As a result, we have:

(A6) $$L_{I_{r}}\left(S\right){|}_{S=T_{P}r^{\alpha }/P_{P}}=\text{exp}\left(-2\pi \left(\lambda_{P}+\lambda_{M}\right)\int_{r>0}\frac{Sv^{-\alpha }}{\mu +Sv^{-\alpha }}vdv\right)$$
